# Metabolic syndrome and the skin: a more than superficial association. Reviewing the association between skin diseases and metabolic syndrome and a clinical decision algorithm for high risk patients

**DOI:** 10.1186/s13098-018-0311-z

**Published:** 2018-02-21

**Authors:** Ellie C. Stefanadi, Georgios Dimitrakakis, Christos-Konstantinos Antoniou, Dimitrios Challoumas, Nikita Punjabi, Inetzi Aggeliki Dimitrakaki, Sangeeta Punjabi, Christodoulos I. Stefanadis

**Affiliations:** 1 Dermatology Department, Athens Medical Centre, Athens, Greece; 2 Heart Surgery Department, University Hospital of Wales, Cardiff, UK; 3 National and Kapodistrian University of Athens and Athens Heart Center, Athens, Greece; 4 Great Ormond Hospital for Children Orthopaedic Unit, London, UK; 5 Saint George Medical School, London, UK; 6 National and Kapodistrian University of Athens and Metropolitan Hospital, Athens, Greece; 7 Dermatology Department, Northwick Park and Middlesex Hospitals, London, UK; 8 National and Kapodistrian University of Athens and Athens Medical Center, Papadiamantopoulou 20, Ilisia, Athens, Greece

**Keywords:** Metabolic syndrome, Skin disease, Psoriasis, Acne

## Abstract

There is ongoing scientific interest regarding comorbidities associated with the metabolic syndrome (MeTS). MeTS comprises a combination of parameters that predispose individuals to the development of type 2 diabetes and cardiovascular disease (CVD). Three or more of the following criteria are necessary: fasting glucose > 110 mg/dl (5.6 mmol/l), hypertriglyceridemia > 150 mg/dl (1.7 mmol/l), HDL levels < 40 mg/dl (men)/< 50 mg/dl (women), blood pressure > 130/85 mmHg, waist circumference (values for Mediterranean populations > 94 cm (men)/> 89 cm (women). In this review we attempted to summarize relevant data by searching dermatological literature regarding associations between various skin conditions and MeTS. A multitude of studies was retrieved and a further goal of the present article is to present plausible mechanistic connections. The severity of skin conditions like psoriasis has been linked with MeTS. Parameters of MeTS like insulin resistance are present in patients with early onset androgenic alopecia, hidradenitis suppurativa acne and rosacea. Since MeTS can lead to CVD and type 2 diabetes early detection of patients would be very important. Also therapeutic intervention on MeTS could lead to improvement on the severity of skin conditions. This reciprocal relationship between skin diseases and MeTS in our opinion holds great interest for further investigation.

## Background

There is ongoing scientific interest regarding comorbidities associated with the metabolic syndrome (MeTS). MeTS [1] comprises a combination of parameters that predispose individuals to the development of type 2 diabetes (T2DM) and cardiovascular disease (CVD). It was first described in 1988 by GM Reaven as syndrome X [2] with various definitions having been proposed since. However, there are still controversies and debate [3] regarding the definition and effective clinical application of MeTS diagnosis, with many studies attempting to correlate it with various organ system pathologies.

In fact any pathophysiologic dysfunction that results in a loss of metabolic control in the body can result in cutaneous disease. There is the notion that a cascade of reactions happens when there is a single hormonal imbalance as it is the case with insulin, and this affects other organ systems [4–6]. Also several studies have shown the existence of increased numbers of oxidative stress and inflammatory markers in various skin diseases something that we observe on the metabolic syndrome as well [7, 8] Table 2.

In this review we attempted to summarize relevant data by searching dermatological literature regarding associations between various skin conditions and MeTS. A multitude of studies was retrieved and a further goal of the present article is to present plausible mechanistic connections.

## Metabolic syndrome: history and definitions

Reviewing the history of MeTS, Professor GM Reaven was the first to describe it as (then) Syndrome X in 1988. It was defined as the condition where individuals with hyperinsulinemia/insulin resistance are disproportionately glucose intolerant, with concomitant dyslipidemia characterized by a high plasma triglyceride (TG) and low high-density lipoprotein (HDL) concentration, and an increase in blood pressure (BP). Notably, all these changes increase cardiovascular disease (CVD) risk [9].

Ten years later the world health organization (WHO) proposed a modified definition. Patients needed to be insulin resistant or to have either T2DM, impaired glucose tolerance (IGT, defined as a glucose level above a predetermined cutoff, commonly 140 mg/dl, for 120 min after ingestion of 75 g of glucose load during an oral glucose tolerance test) or impaired fasting glucose more than 100 mg/dl (IFG). Euglycemic hyperinsulinemic clamp studies or Homeostatic model assessment (HOMA-IR) could be used as evidence of insulin resistance. In addition, two of the following criteria should also be met: obesity (abdominal or overall), dyslipidemia (high TG or low HDL) concentration, elevated blood pressure, or microalbuminuria [10]. The above definition finds mandatory the insulin resistance criterion but allows T2DM to be diagnosed with metabolic syndrome if they meet two of the rest of the criteria. Because some of the measurements are not performed routinely, for example, euglycemic clamp studies (for insulin resistance measurement), this definition is not easily applied in every day clinical practice.

In 1999, the European Group for the Study of Insulin Resistance (EGIR) proposed a modification to the WHO definition [11]. This time a fasting plasma insulin value that is greater than the 75th percentile is the main criterion for the syndrome something that simplified the diagnosis excluding however patients with T2DM. Also two of the following criteria need to be met: obesity, hypertension and dyslipidemia. The obesity criteria were simplified to waist circumference, whereas the WHO definition used a choice of waist-to-hip ratio or body-mass index. Microalbuminuria was excluded as a diagnostic criterion.

In 2001, the National Cholesterol Education Program Adult Treatment Panel (NCEP-ATP) III published their criteria for diagnosing MeTS. It is a more simple and easy to use definition. Five criteria were specified: abdominal obesity, estimated by an increased waist circumference, high TG and low HDL concentrations, elevated BP, and glucose intolerance [12]. Presence of any three of the above criteria is considered compatible with a MeTS diagnosis according to the NCEP-ATP III.

The International Diabetes Federation (IDF) in 2005 proposed yet another definition that included a definite criterion of waist obesity plus two of the other components. It should be mentioned though that today non-obese patients are also diagnosed with MeTS (termed metabolically obese). The basis for this lies in the fact that this group has increased visceral fat accumulation [12].

Due to possible confusion from their contradictory definitions, the IDF and the NCEP-ATP III more recently reached a common harmonized definition comprising five equal criteria: elevated waist circumference (defined according to specific population characteristics), low HDL levels, high TG levels, elevated BP, elevated fasting glucose [11–15]. Once more, presence of at least three was necessary and sufficient for MeTS diagnosis (Table 1).

**Table 1:** Metabolic syndrome definition

	WHO	EGIR 1999	NCEP ATPIII	IDF 2005	Harmonized criteria
Criteria	Insulin resistance IGF, OFG, TDM 2, evidence of IR plus two of the five	Hyperinsulinemia plus two of four	None absolute three of five	Central obesity obligatory	Three or more
Obesity	Waist/hip ratio > 0.90 M > 0.85 F or BMI > 30 kg/m^2^	Waist circumference > 94 cm M > 80 cm F	Waist circumference > 40 in. M > 35 in. F	Waist circumference > 94 cm M > 80 cm F	Waist circumference > 90 cm M > 80 cm F
Hyperglycemia	Insulin resistance	Insulin resistance	Fasting glucose > 100 mg	Fasting glucose > 100 mg	Fasting glucose > 5.6 mmol/l or T2DM
Dyslipidemia	Triglycerides > 150 mg/dl HDL < 35 mg/dl M < 39 mg/dl F	TG > 177 mg/dl HDL < 39 mg/dl	TG > 150 mg/dl	TG > 150 mg/dl	TG > 1.69 mmol/l or on previous antilipidemic treatment
Dyslipidemia seperate			HDL < 40 mg/dl M < 50 mg/dl	HDL < 40 mg/dl M <50 mg/dl	HDL < 1.03 mmol/l M < 1.29 mmol/l F
Hypertension	> 140/90 mmHg	> 140/90 mmHg	> 130/85 mmHg	> 130/85 mmHg	> 130/85 mmHg or known hypertension
Other	Microalbuminemia				

The previous data serves as a basic introduction to the history of MeTS. Of note, several authors argue that all risk factors should be treated equally and that use of the term MeTS in routine clinical practice should be done cautiously. It has also been suggested that MeTS could be considered a pathophysiologic mechanism/continuum rather than a syndrome and be graded according to severity and potential for CVD occurrence [14].

## Metabolic syndrome pathophysiology

The most widely accepted theory for the pathophysiology of the syndrome is insulin resistance [16]. The muscle, fat and liver cells do not respond properly to hormone insulin and cannot absorb easily glucose from the bloodstream. Beta cells of the pancreas initially try to produce more insulin to achieve euglycemia. Progressively, pancreas fails to keep up with the increased demand for insulin and excess glucose builds up in the bloodstream [16].

### Increased waist circumference

Increased adipose tissue plays a major role in the development of insulin resistance. Visceral obesity increases the amount of free fatty acids (FFA) in the body, and FFAs decrease insulin-mediated glucose uptake at the muscle-cell level. As a result, blood glucose levels increase requiring higher levels of insulin secretion insulin in. In the liver, FFA increase the production of glucose, TG and very low density lipoproteins. Moreover, insulin itself has a lipolytic effect—thus a vicious circle develops where increased levels of insulin lead to increased lipolysis, in turn increasing FFA levels and further promoting insulin resistance and stimulation of its production and secretion [16].

### Triglycerides, HDL, LDL

The aforementioned increase in TG levels following the increase on FFA’s is a good indicator of MeTS presence. In addition to that HDL decreases caused by decreases in the cholesteryl ester content of lipoprotein core. LDL levels increase as well [16, 17].

### Hyperglycemia

The constant need for insulin production eventually reaches non-sustainable levels for the pancreas and insulin levels start to drop—it is at that point that hyperglycemia ensues. Constantly increased levels of FFA can also act in a toxic way on the beta cells and suppress insulin production [16].

### Blood hypertension

The hypertensive effect of MeTS has been attributed to the lack of the vasodilatory effect of insulin—insulin resistance also affects vasodilation. Concomitantly, insulin increases sodium absorption by the kidneys, while FFA have vasoconstrictive effects. Finally, increased levels of insulin result in increased sympathetic nervous system activity and the above in tandem lead to the development of hypertension.

### MeTS coronary artery disease and diabetes

The aforementioned risk factors are similar risk factors for coronary artery disease and diabetes mellitus. Although MeTS is a different entity from CAD it appears that on both Mets and DM we get coronary microvascular disease as an early culprit [17]. The state of chronic inflammation and the raised lipids can explain in some extend the fact. Also, MeTS is considered to act in a toxic way on various organ systems including b-cells on pancreas. Thus the abdominal accumulation of fat, but also the other criteria of the syndrome can evolve to DM mainly type 2 [18].

Overall MetS predisposes individuals to DM or CAD. Wilson et al. showed that in a group of patients with MeTS one-third developed CAD and half of the patients developed DM type 2 over a period of 8 years follow-up [19].

Insulin resistance is accompanied by other pathophysiologic disturbances, some requiring further elucidation. Such are increases in proinflammatory cytokines, prothrombotic factors, homocysteine, serum viscosity, leptin and resistin levels, a decrease in adiponectin, occurrence of non-alcoholic fatty liver disease and polycystic ovarian syndrome. This fact could become a base for further investigation of the syndrome in relation to other diseases and in particular dermatological diseases [1, 11, 13].

### MeTS and the skin

Thus, it appears that dis regulation of skin physiology can predispose to MeTS and vice verse [20].

Any pathophysiologic dysfunction that results in a loss of metabolic control in the body can result in cutaneous disease. The fat accumulation in MeTS with the progressive development of insulin resistance induces a cascade of hormonal changes, such as the effects on growth hormone. Hormones follow a principal of autoregulation in a synergistic manner. Thus, deterioration of androgen-dependent skin diseases like acne or androgenic alopecia are to be expected. At the same time, inflammatory markers like TNF-α, IL-17, IL-23 and oxidative stress appear involved in many autoimmune and inflammatory skin conditions [21–23] that are certainly present in both conditions. Consequently, it is not a paradox to suspect common pathophysiology pathways [4, 5, 11].

## Metabolic syndrome and psoriasis

Amongst the various skin conditions investigated, psoriasis holds a great interest in relation to MeTS. Psoriasis is a chronic inflammatory disease, affecting 1–4% of the general population, considered a multisystem disorder, rather than a skin disease alone. The prevalence of the MeTS has been estimated to be about 15–25% in the general population, appearing significantly higher (an increase by about threefold) in psoriatic patients, as documented by many case–controls studies [24, 25].

The association between psoriasis and MeTS is directly correlated with the severity of psoriasis. Various population based studies from all continents show that MeTS is an independent comorbidity in psoriatic patients. In 1978 McDonald et al. investigated connection of psoriasis with vascular disease and Gisondi et al. found a prevalence of 30.1% of MeTS in psoriasis patients vs 20.6% in control [26, 27]. The cross–sectional study of Cohen et al. also showed an association between psoriasis and MeTS [28]. The study of Praveenkumar et al. suggests that MeTS, as well as dyslipidemia (low HDL), are more common in psoriasis patients (MeTS prevalence in psoriasis 60% vs 40% in controls, p = 0.12). In particular, risk factors such as dyslipidemia, increased BP, obesity, insulin resistance were more prevalent in psoriatic patients, although differences did not reach statistical significance [29]. Another study from Thailand conducted by Kokpol et al. showed that all psoriatic patients had higher prevalence of MeTS than the general population (49.25% vs 30.65%) [30]. The metabolic components which were significantly higher in the cases than controls included hyperglycemia, high blood pressure (HBP) and abdominal obesity. Studies in a large population from Henseler et al. showed similar results, with systemic disorders, such as diabetes and obesity occurring significantly more often in patients with psoriasis than in control subjects [31]. Itani et al. investigated 150 patients with psoriasis, and noted a twofold increase in MeTS in relation to controls (35.3% vs 18.0%, p < 0.001) and even higher in cases of inverse psoriasis and nail pitting [32]. Meziane et al. also found a higher incidence of MeTS among 150 psoriatic patient [33]. An indirect relation also has to be considered, given that psoriatic male patients are more often smokers thus more susceptible to MeTS-related disorders. Owczarczyc-Suczonek et al. found, in a group of 69 psoriatic patients, that the prevalence of MeTS was 25.81% vs 21.02% in the control group (p = NS), the mean HOMA-IR (insulin resistance index) being 1.93 vs 1.94 (p = NS) [34]. It has also been reported that liver fibrosis due to fat accumulation is more frequent in comparison to the general population [35].

Neimann et al. investigated cardiovascular risk factors in patients with mild and severe psoriasis. CAD risk factors where more prevalent in patients with severe psoriasis [24]. A plausible mechanistic hypothesis postulates that psoriasis predisposes to MeTS and arterial stiffness due to the various inflammatory markers circulating in the blood, the so-called ‘psoriatic march’. The hypothesis states that psoriasis is a chronic systemic inflammatory disease resulting in insulin resistance by down-regulation of insulin receptors. Cytokines of the Th1 pathway [interferon-γ, interleukin (IL)-2, IL-12, and tumor necrosis factor (TNF)-α] predominate in both psoriatic and atherosclerotic plaques [21]. In addition, the decrease in the expression of insulin receptors in endothelial cells results in reduction of nitric oxide (NO), a vasodilatory agent. Thus, vasoconstriction ensues, leading to increased arterial stiffness. As a result, increased incidence of myocardial infraction (MI) and stroke has been reported. It has been shown that the use of an insulin stimulating agent (glucagon like peptide 1—GLP-1) can improve psoriasis emphasizing the effect of insulin resistance in inflammation [25, 35, 36]. There are successful attempts to prove that various inflammatory markers increase initially on the skin and then systemically on psoriasis as C reactive protein. This markers can deteriorate the metabolic syndrome. Also oxidative stress and DNA/RNA damage is increased in patients with psoriasis and metabolic syndrome. So, the evidence shows at least similarities on the pathophysiology of both conditions [37]. Chronic inflammation of psoriasis and the downregulation of insulin receptors predisposes to MeTS. The chronic inflammation of MeTS affects the homeostasis of the skin in combination with genetic and other factors. A late article for Korkmaz et al. states that a change in expression of apoptosis activators may contribute to the development of MeTs in patients with psoriasis [38].

Screening of psoriatic patients for CVD risk factors may uncover those both at high risk and suboptimally managed [39]. Recent algorithm for patients with psoriasis and metabolic syndrome has been published by Radtke et al. [40]. According to the algorithm patients with psoriasis and metabolic syndrome should be referred for further management when they develop three of the following criteria: visceral obesity (waist circumference > 94 cm in men or > 80 cm in women), triglycerides > 150 mg/dl or on specific therapy, HDL < 40 mg/dl in men or < 50 mg/dl in women or on specific treatment, systolic pressure > 130 mmHg or diastolic > 85 mmHg or on treatment, fasting plasma glucose > 100 mg/dl. Follow up should be arranged every 6 months for severe psoriasis (or for those on systemic medication), or annually for mild cases. Furthermore, lifestyle changes, such as smoking and alcohol cessation, dietary habits and exercise should be pursued.

Overall psoriasis may be independently associated with the development of MI and ischemic heart disease [22]. Conflicting studies do not prove always such a relation. There are however populations differences on the severity of obesity on Western society in comparison to other populations possibly explaining this results [41]. Consequently, physicians should be aware of an increase in CVD risk in psoriatic patients and adjust their management accordingly [24, 35, 36, 42–49].

## Sebaceous and apocrine glands disorders

### Acne

Acne, a disorder of the pilosebaceous unit, is a multifactorial condition. Endocrine abnormalities can produce acne as it occurs in women with polycystic ovary syndrome, which has itself been related to MeTS. Recently, Nagpal and colleagues conducted a cross-sectional study in 100 post adolescent male patients [50, 51]. They found a statistically significant increase in the incidence of insulin resistance and MeTS prevalence in their patient group in comparison with the control group in 100 post-adolescence male patients. Del Prete and colleagues investigated 32 male individuals with acne concluding that parameters of MeTS, mainly insulin resistance, were higher in the patient group compared with controls [52]. Acne is a multifactorial disorder. A well-known contributing factor is the excess on testosterone levels. Patlolla et al. showed that obese/insulin resistant patients with polycystic ovary syndrome (PCO) have low SHBG and hence an increased fraction of free testosterone [53]. The PCO syndrome is characterized of insulin resistance, obesity, acne, hirsutism. Thus one of the possible pathophysiologic mechanism explaining deterioration of acne in MeTS could be the increase on free testosterone.

The pilosebaseus unit appears to play an important role as an antimetabolic syndrome factor. The secretion of sebum decreases the lipid levels. At the same time disregulation of the unit it self can predispose to the expression of metabolic syndrome. This vise versa relationship hold great interest for future investigation [20].

### Rosacea

A complex disorder of sebaceous glands and blood microcirculation in the face, rosacea has been investigated in relation to MeTS. In particular, the study by Akin Belli et al. showed a relationship between rosacea and CVD risk factors [54]. Dysregulation of Sympathetic system, testosterone effect and blood hypertension appear on cases of Roseacea and MeTS [16].

### Hidradenitis suppurativa

The apocrine gland disorder hidradenitis suppurativa (HS) affects body part areas such as the axillae and groin. Study results highlight the high comorbidity burden of patients with HS compared with matched control subjects. Among other, there was a higher incidence of diabetes, obesity, dyslipidemia and hypertension. A meta-analysis by Tzellos et al. found a significant relation of MeTS and HS [48]. Unsurprisingly, recent studies show that patients with HS may have one or more MeTS components despite young age and target screening is currently advised [55–57]. Also, one possible pathophysiologic mechanism could be the effect of androgens. Often the condition improves with oral anti androgen medication [58].

### Alopecia

Apparently, androgenic alopecia (AGA) has been investigated in relation to MeTS [59]. Some studies show that early onset of AGA could be an independent factor related to the syndrome. In 2000 Matilainen et al. observed early AGA associated with increased insulin resistance [60]. A case control study of 100 young male patients conducted by Banger et al. detected a statistically significant difference regarding the number of individuals in the group of AGA fulfilling the criteria for MeTS in comparison with the control group [61]. The study by El Sayed et al. among 90 female patients with AGA showed a statistically significant increase in the incidence and severity of female pattern hair loss especially in relation to hypertension and obesity (defined by NCEP-ATP III criteria) [62]. In contrast, another study from Ozbas Gok et al. did not prove a relation between AGA and MeTS apart from a difference in systolic pressure between disease-based groups [63]. Again the increase on free testosterone on insulin resistance syndromes can be a contributing factor on the deterioration of AGA. Oxidative stress mechanism in addition come to accelerate aging process as AGA deteriorates with age. Alopecia areata (AA) is a non-scaring form of alopecia of autoimmune etiology. There are case reports of AA with underlying MeTS, suggesting a possible field of future investigation [64].

## Inflammatory and autoimmune skin disease

### Lichen planus

Lichen planus patients are at increased risk for CVD as it has been shown that they develop dyslipidemia more frequently. Moreover, patients with lichen planus were found to have higher markers of both metabolic and cardiovascular risk factors in relation to controls, most probably due to long standing inflammation associated with the condition [65, 66].

### Cutaneous manifestations of systemic lupus erythematosus

In systemic lupus erythematosus, adipokines could also play a role in the occurrence of MeTS. A case report from Sato et al. implies that insulin resistance type Β (severe resistance caused by polyclonal IgG antibodies directed against insulin receptors) should be suspected in patients with systemic lupus and hypoglycemia (induced by simultaneous release of receptors) [67].

### Atopic dermatitis

Atopic dermatitis is positively related to MeTS adding to the panel of inflammatory skin conditions with systemic involvement. Animal models with AD were found having a higher incidence of liver fat accumulation [68]. Atopic dermatitis is being investigated in relation to MeTS on a similar basis as psoriasis. The co-existence of both skin conditions and metabolic syndrome is being refereed as the “inflammatory skin march” [69]. One possible common feature on both entities is the alteration on expression of T cell cytokines especially Tc 2 derived cytokines. The TH2 subset of T helper lymphocytes are recruited on the dermis and epidermis in atopic dermatitis on large amounts [70].

### Seborrheic dermatitis and other inflammatory skin conditions

This very common skin condition affecting mainly scalp and areas of the face like the eyebrows and nasolabial folds appears to be on recent studies a predictive factor for MeTS [41].

Most recently, a study was published in a Brazilian population, relating MeTS with the autoimmune skin disease pemphigus but the chronic use of steroid treatment has to be considered as a potential confounder [71].

Chronic urticaria shares some common pathophysiology with MeTS as well [69].

Overall the production of adipokines by fat cells in MeTS, like leptin, adiponectin, TNF-α, IL-6, monocyte chemotactic protein-1 (MCP-1), and others, now recognized as a part of the innate immune system, has an important role in the pathogenesis of insulin resistance. In addition, they may affect accumulation of leukocytes on tissues and predispose to CVD. Medical treatments that improve skin conditions, including methotrexate, decreased inflammatory markers and the incidence of coronary disease [72]. Table 2 lists proinflammatory molecules associated with various inflammation-related conditions discussed in this article.

**Table 2:** Inflammatory markers in MeTS and skin-related conditions

Metabolic syndrome	IL-4, IL-6, IL-7, IL-8, IL-9, IL-10, G-CSF, TNF-α, VEGF, PDGF-BB, GM-CSF, RANTES
Psoriasis	IL-1, IL-2, IL-6, IL-12, IL-15, IL-22, IL-23, IFN-γ, TNF-α
Lichen planus	IP-10, MCP-1, RANTES, MIG
Atopic dermatitis	IL-4, IL-5, IL-12, IL-13, IL-16

*IL* interleukin, *G-CSF* granulocyte-colony stimulating factor, *TNF-α* tumor necrosis factor-α, *VEGF* vascular endothelial growth factor, *PDGF-BB* BB isoform of the platelet derived growth factor, *GM-CSF* granulocyte-macrophage colony-stimulating factor, *RANTES* regulated on activation, normal T cell expressed and secreted (chemokine, also known as CCL-5), *IFN-γ* interferon-γ, *IP-10* interferon gamma-induced protein 10, *MCP-1* monocyte chemoattractant protein 1, *MIG* monokine induced by gamma interferon

## Cutaneous tumors

In 2012 Nagel et al. investigated the relationship of MeTS parameters with skin cancer. A positive relation was found in women with malignant melanoma (MM) and increased blood pressure. Also, men with MM tend to have higher body mass indices (BMI). Women with squamous cell carcinoma/non melanoma skin cancer had a tendency towards increased glucose and TG. Moreover, development of xanthelasmata and tendon xanthomas (although obviously not proliferative, tumor-like disorders) is quite typical of congenital hyperlipoproteinemias [73–75].

## Skin aging

Nagase et al. investigated the skin aging process in animal models with MeTS. They found evidence of both oxidative stress and upregulated inflammatory markers as well as increased expression of mineralocorticoid receptors in the skin [76]. They propose a similar mechanism of aging to the one adopted for internal organs in MeTS. More specifically, oxidative stress and inflammation are related to MeTS, consequently, free oxygen species damaging DNA, mitochondrial function and producing hormonal dysregulation (including insulin resistance) have been associated with the aging process [77]. It appears that skin collagen glycation is related to both MeTS parameters and aging.

Glycation products cross-link with collagen bundles interfering with its function. This phenomenon becomes more intense in diabetics, accelerating skin aging. Intrinsic factors like the Maillard reaction but also oxidative stress affect fibroblast gene expression reducing levels of metalloprotease inhibitors and increasing production of metalloproteases that degrade collagen. Moreover, collagen itself becomes stiffer with an altered function. Based on this theory, many studies propose antioxidants and inhibitors of collagen glycation as preventive treatments. This condition, along with oxidative stress, inflammation and endothelial dysfunction, has been studied in relation to diabetes. Antidiabetic medication for patients with T2DM would theoretically reduce the reactions’ extend by controlling blood glucose levels [78]. In general, collagen glycation is considered a platform for research on the pathogenesis of skin aging [79, 80]. Skin autofluorescence showed to be effective on detection of individuals with advanced glycation end products and predisposition to MeTS. This could be a promising method for research and future population studies [19].

## Miscellaneous skin diseases

Conditions such as holoderma (knuckle pads), striae, skin tags, impaired wound healing, vitiligo have been directly or indirectly connected to MeTS [81–86].

## Microscopic changes

Investigators have shown interest in the microscopic changes to the skin structure in individuals with MeTS. Janovska and colleagues microscopically observed dyspigmentations and telangiectasias on the skin of patients. Moreover, histological sections showed dermal elastosis, thickening of stratum spinosum and basal membrane, acanthosis, as well as mild Τ lymphocytic infiltration around capillaries. Bcl-2 anti-apoptotic protein was accumulated in the epidermis more significantly in participants with MeTS [87].

Furthermore, Puchau et al. found an inverse correlation between several trace element levels (including zinc, selenium and copper) from nail samples of individuals and blood inflammatory cytokine levels, stressing the importance of these antioxidant elements against chronic inflammatory states and skin aging [88].

Table 3 summarizes studies exploring associations between MeTS and various skin diseases.

**Table 3:** Studies of metabolic syndrome and skin disease

First author	Year	Skin condition
Praveenkumar et al. [29]	2016	Psoriasis
Kokpol et al. [30]	2014	Psoriasis
Henseler et al. [31]	1995	Psoriasis
Owczarczyk-Saczonek et al. [34]	2015	Psoriasis
Gisondi et al. [26]	2007	Psoriasis
Nagpal et al. [51]	2016	Acne
Del Prete et al. [52]	2012	Acne
Akin Belli et al. [54]	2016	Rosacea
Shlyankevich et al. [56]	2014	Hidradenitis suppurativa
Banger et al. [61]	2015	Androgenic alopecia
El Sayed et al. [62]	2016	Androgenic alopecia
Saleh et al. [66]	2014	Lichen planus
Arias-Santiago et al. [65]	2011	Lichen planus
Lee et al. [68]	2017	Atopic dermatitis
Ambiel et al. [71]	2014	Pemphigus
Saylam Kurtipek et al. [83]	2015	Holoderma
Wali et al. [84]	2016	Striae
Chen et al. [85]	2016	Acanthosis nigricans
Puttegowda et al. [75]	2015	Tendon xanthomas
Nagase et al. [76]	2013	Skin aging
Penington et al. [86]	2007	Wound healing

## Conclusions

MeTS includes the following parameters: Increased waist circumference, high TG and low HDL level, increased blood pressure, and increased blood fasting glucose. The base of its pathophysiology lies in insulin resistance. Individuals with the syndrome seem to have increased incidence of CVD and T2DM [17, 89]. Application of this observation in every day clinical practice means that a higher level of care should be applied in such patients with closer monitoring and aggressive treatment of each individual parameter. Monitoring risk factors like xenobiotics and genetics is a logic approach. Also, lifestyle factors like nutrition and exercise can be part of a prevention program [18].

MeTS and the skin is still a field of active research and associations are being established with other medical conditions. We found several studies investigating psoriasis, and other inflammatory skin conditions in conjunction with MeTS. There were also attempts to associate MeTS with skin aging, skin neoplasias and skin autoimmune diseases. These observations open novel pathways while trying to elucidate the pathophysiology of skin disease and more importantly introduce and evaluate novel yet mechanistically sound treatments, e.g. insulin stimulating factor improving psoriasis.

Patients with psoriasis have a higher incidence of metabolic syndrome parameters and are at increased risk of CAD [25]. Patients with other inflammatory skin conditions like atopic dermatitis have derangement in several metabolic syndrome parameters [69]. Inflammatory and autoimmune skin conditions like lichen planus are related to chronic inflammation and MeTS [65]. A positive correlation has been found for pemphigus and systemic lupus [71]. Individuals with acne are more prone to have insulin resistance [50]. So, far routine investigation on metabolic parameters takes place only for patients on systemic medication for skin disease (isotretinoin, methotrexate, azathioprine, ciclosporine, prednizolone) [90]. Routine tests for metabolic parameters could be applied in the future Table 4.

**Table 4:** Proposed management of patients with skin disease at higher risk for CVD

Skin condition	Risk factor for CVD	Management
Psoriasis	Severity, nail pitting	Refer if ≥ 3 of: TG > 150 mg/dl, HDL < 40 mg/dl, waist circumference > 94 cm (males)/> 80 cm females, fasting plasma glucose > 100 mg/dl
Androgenic alopecia	Early onset in males, severity in females	Consider screening for insulin resistance
Acne-rosacea	Adults	Treat insulin resistance
Atopic dermatitis	Female sex	Consider abdominal ultrasound for visceral fat accumulation
Lichen planus	Degree of inflammation (?)	Manage dyslipidemia/CVD
Hidradenitis suppurativa	Severity	Check for MeTS parameters even in young patients
Squamous cell carcinoma	Female sex	Blood glucose, triglyceride levels monitoring
Melanoma	Male sex	Manage hypertension
Skin tags, acanthosis nigricans, impaired wound healing	Undetermined	Treat insulin resistance
Aging	Collagen glycation	Tighter glycemic control

Also the clinical presentation of a patient with multiple skin tags, acanthosis nigricans, granuloma anulare or necrobiosis lipoidica is already related to T2DM. Metabolic parameters deterioration was observed in patients with HS and rosacea [54]. Moreover, AGA in both sexes had an earlier onset and severity in MeTS [59]. Patients with skin cancer malignant melanoma suffer more often from blood hypertension with a positive relation also found for squamous cells carcinomas [73]. Microscopic changes were observed on histology sections while oxidative stress and inflammation affect the skin as well [87]. The result is the skin aging. Whether this is comparable to premature aging as in diabetes with the effects of glycation remains to be further investigated [19] Table 3.

An interesting aspect of the skin function has been investigated in relation to its ability to eliminate from the body xenobiotics (drugs, pesticides and other chemical compounds), remove free radical species by having an antioxidant capacity and even reduce cholesterol levels by the excretory function of sweat and sebaceous glands [20].

More specifically, the skin expresses all the enzymes of the xenobiotic eliminating systems like cytochrome P450 enzymes, flavin-dependent monooxygenase, monoamine oxidase, alcohol dehydrogenase, and aldehyde dehydrogenase. In addition to that, it is equipped with drug metabolizing systems and antioxidant enzymes. Sweat is found to contain a high quantity of toxic substances. Moreover, sebum clears excess cholesterol [20]. It is a well-known notion that isotretinoin that reduces sebum production is related to increased levels of cholesterol in the body. The important function of skin as an insulin regulator unfortunately comes to surface in severe burns where patient can develop a long term insulin resistance [78].

Based on the above, a higher level of suspicion should exist regarding skin disease patients in primary and secondary care in order to achieve earlier detection of those in high risk of CVD or T2DM [20]. Presence of the aforementioned skin conditions in people meeting MeTS criteria appears not to be uncommon. In our opinion, this observation could be interpreted in the following ways:

- Firstly, it once more emphasizes the interconnection among organ systems and the extent to which the skin is involved in metabolic disorders.
- Secondly, results from the increasing interest and related research suggest a potential for early detection and better management of people at risk for CVD or T2DM (Table 4). Skin involvement and its extent could also assist in interpreting some of the residual (i.e. not explainable by conventional CVD risk factors) variability in CVD occurrence.

The idea of a simple skin test that could in the future give information about the metabolic status of a patient is appealing and would undoubtedly lead to improvement of care for our patients [19].

## Abbreviations

IL: interleukin
G-CSF: granulocyte-colony stimulating factor
TNF-α: tumor necrosis factor-4 α
VEGF: vascular endothelial growth factor
PDGF-BB: BB isoform of the platelet derived 5 growth factor
GM-CSF: granulocyte-macrophage colony-stimulating factor
RANTES: regulated on activation, normal T cell expressed and secreted (chemokine, also known as CCL-5)
IFN-γ: interferon-γ
IP-10: interferon gamma-induced protein 10
MCP-1: monocyte 8 chemoattractant protein 1
MIG: monokine induced by gamma interferon
MeTS: metabolic syndrome
T2DM: type 2 diabetes mellitus
CVD: cardiovascular disease
HDL: high density lipoprotein
TG: triglycerides
BP: blood pressure
WHO: World Health Organisation
IGT: impaired glucose tolerance
IFG: impaired fasting glucose
GLP: glucagon like peptide
HOMA: homeostatic model assessment
EGIR: European Group for Insulin Resistance
NCEP-ATP: National Cholesterol Education Program Adult Treatment Panel
IR: insulin resistance
FFA: free fatty acids
DM: diabetes mellitus
IDF: International Diabetes Federation
LDL: low density lipoprotein
NO: nitric oxide
MI: myocardial infraction
AGA: androgenic alopecia
PCO: polycystic ovary
HS: hidradenitis suppurativa
AA: alopecia areata
AD: atopic dermatitis
MCP: monocyte chemotactic protein
MM: malignant melanoma
BMI: body mass index
